# The importance of radial multiplanar reconstructions for assessment of triangular fibrocartilage complex injury in CT arthrography of the wrist

**DOI:** 10.1186/s12891-020-03321-2

**Published:** 2020-05-07

**Authors:** Jan-Peter Grunz, Carsten Herbert Gietzen, Karsten Luetkens, Matthias Wagner, Karlheinz Kalb, Thorsten Alexander Bley, Lukas Lehmkuhl, Jörg van Schoonhoven, Tobias Gassenmaier, Rainer Schmitt

**Affiliations:** 1 Department of Diagnostic and Interventional Radiology, Rhön-Klinikum Campus Bad Neustadt, Von-Guttenberg-Str. 11, 97616 Bad Neustadt an der Saale, Germany; 2 Department of Diagnostic and Interventional Radiology, University Hospital Würzburg, Oberdürrbacher Str. 6, 97080 Würzburg, Germany; 3 Department of Hand Surgery, Rhön-Klinikum Campus Bad Neustadt, Von-Guttenberg-Str. 11, 97616 Bad Neustadt an der Saale, Germany

**Keywords:** Triangular fibrocartilage, Wrist, Arthrography, Tomography, X-ray computed

## Abstract

**Background:**

Triangular fibrocartilage complex (TFCC) lesions commonly cause ulnar-sided wrist pain and instability of the distal radioulnar joint. Due to its triangular shape, discontinuity of the TFCC is oftentimes difficult to visualize in radiological standard planes. Radial multiplanar reconstructions (MPR) may have the potential to simplify diagnosis in CT wrist arthrography. The objective of this study was to assess diagnostic advantages provided by radial MPR over standard planes for TFCC lesions in CT arthrography.

**Methods:**

One hundred six patients (49 women, 57 men; mean age 44.2 ± 15.8 years) underwent CT imaging after wrist arthrography. Two radiologists (R1, R2) retrospectively analyzed three randomized datasets for each CT arthrography. One set contained axial, coronal and sagittal planes (MPR_Standard_), while the other two included an additional radial reconstruction with the rotating center either atop the ulnar styloid (MPR_Styloid_) or in the ulnar fovea (MPR_Fovea_). Readers evaluated TFCC differentiability and condition. Suspected lesions were categorized using Palmer’s and Atzei’s classification and diagnostic confidence was stated on a five-point Likert scale.

**Results:**

Compared to standard planes, differentiability of the superficial and deep TFCC layer was superior in radial reconstructions (R1/R2; MPR_Fovea_: *p* < 0.001; MPR_Styloid_: *p* ≤ 0.007). Palmer and Atzei lesions were present in 86.8% (92/106) and 52.8% (56/106) of patients, respectively. Specificity, sensitivity and accuracy for central Palmer lesions did not differ in radial and standard MPR. For peripheral Atzei lesions, sensitivity (MPR_Standard_ 78.6%/80.4%, MPR_Styloid_ 94.6%/94.6%, MPR_Fovea_ 91.1%/89.3%) and accuracy (MPR_Standard_ 86.8%/86.8%, MPR_Styloid_ 96.2%/96.2%, MPR_Fovea_ 94.3%/93.4%) improved with additional styloid-centered (*p* = 0.004/0.008) and fovea-centered (*p* = 0.039/0.125) reconstructions. No substantial difference was observed between both radial MPR (*p* = 0.688/0.250). Interrater agreement was almost perfect for each dataset (*κ*_Standard_ = 0.876, *κ*_Styloid_ = 0.894, *κ*_Fovea_ = 0.949). Diagnostic confidence increased with addition of either radial MPR (*p* < 0.001).

**Conclusions:**

Ancillary radial planes improve accuracy and diagnostic confidence for detection of peripheral TFCC lesions in CT arthrography of the wrist.

## Background

Ulnar-sided wrist pain and instability of the distal radioulnar joint (DRUJ) can be the result of degenerative, traumatic or combined lesions of the triangular fibrocartilage complex (TFCC) [1, 2].

**Fig. 1:**
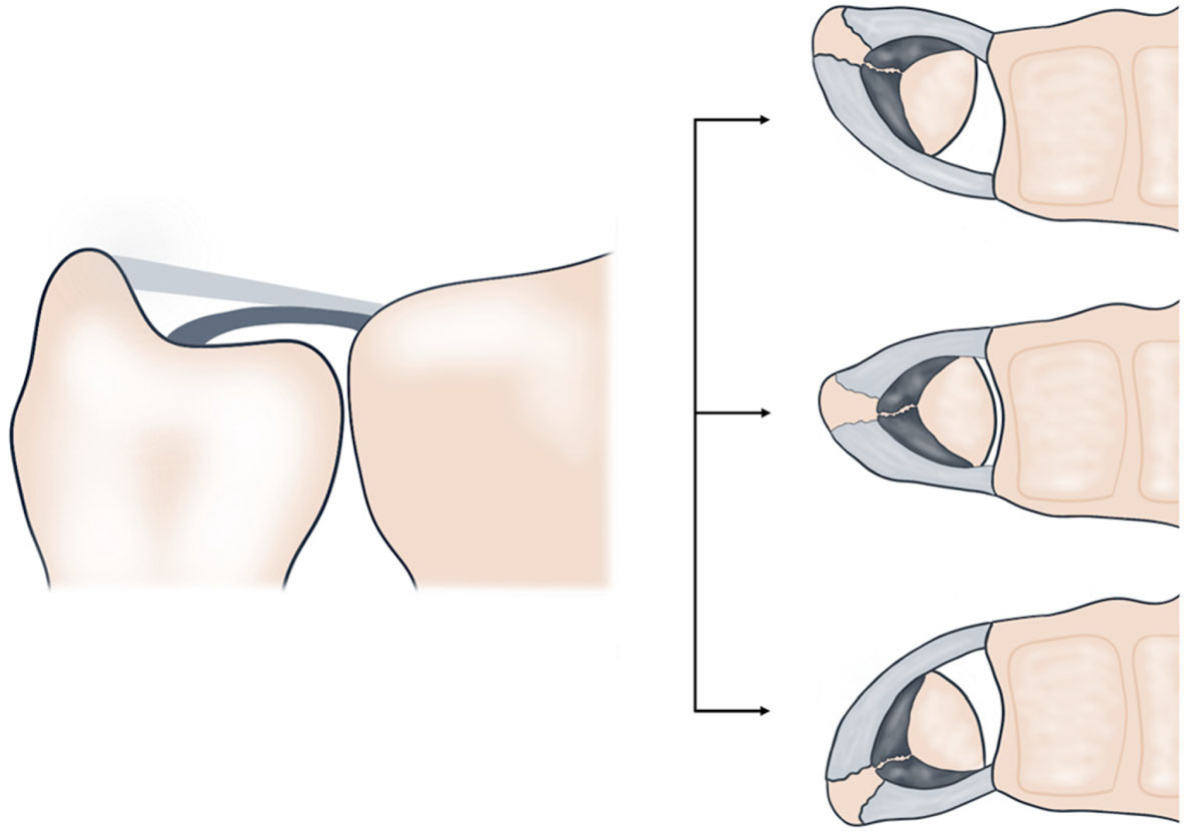
Left side: Schematic coronal display of the triangular fibrocartilage complex (TFCC). While both TFCC layers originate from the sigmoid notch of the radius, the superficial (light grey) and deep (dark grey) layer of the TFCC insert at the ulnar styloid process and ulnar fovea, respectively. Right side: Schematic axial display of the dorsal and palmar radioulnar ligaments during forearm pronation (upper right), neutral position (middle right) and supination (lower right) from an axial perspective

Anatomically, the TFCC comprises of an avascular central disc originating from the articular cartilage of the sigmoid notch of the radius and a vascularized ulnar periphery [3]. The peripheral TFCC consists of two main components and possesses a complex three-dimensional shape: The proximal triangular ligament inserts in the ulnar fovea and represents the deep layer of the TFCC. It is composed of the dorsal and palmar radioulnar ligament which originate directly from the cortex of the distal radius, frame the central disc and tighten oppositely during pro- and supination to maintain stability of the DRUJ [4–6] (Fig. 1). In contrast, the distal hammock structure inserts at the ulnar styloid process and represents the superficial layer of the TFCC. Together with the ulnocarpal ligaments, it supports the transition of axial force from the ulnar-sided wrist to the forearm [7, 8].

TFCC lesions are traditionally categorized according to the classification of Palmer [9], discerning traumatic (class 1) and atraumatic (class 2) causes. However, several aspects limit this approach: Firstly, distinguishing between traumatic and pre-existing degenerative lesions is not always possible. Secondly, the border between central and peripheral lesions is not exactly defined. Finally, and most important, the deep and superficial layer of the ulnar TFCC are not differentiated. Except for rare ulnocarpal ligament injury (Palmer 1C), all peripheral lesions are considered Palmer 1B despite requiring different therapeutic approaches [10].

In 2011, Atzei and Luchetti [11] introduced a new classification system that particularly respects the ulnar-sided TFCC anatomy and treatment options for different lesions. While isolated superficial layer discontinuity (Atzei 1) does not diminish DRUJ stability, unstable deep layer lesions (Atzei 3) require refixation [12, 13]. Patients benefit greatly from the non-surgical differentiation between stable and unstable Palmer 1B lesions. However, radiologists face the challenging task to evaluate the delicate ulnar periphery of the TFCC with even more precision to estimate whether a lesion requires surgery. Further limiting the visibility of lesions, clinical experience shows that the three radiological standard planes (axial, coronal and sagittal) frequently fail to fully depict the complex shape of the TFCC. Although not routinely used in the initial evaluation of ulnar-sided wrist pain [14], CT and MR arthrography are considered the imaging reference for TFCC injuries [15–18]. Studies on angulated multiplanar reconstructions (MPR) have shown improved diagnostic confidence in MRI and CT arthrography of the wrist [19–21] but the advantages of radial MPR for TFCC depiction are not well established in the literature. With this work, we aim to assess the benefits of radial MPR in CT wrist arthrography regarding diagnostic accuracy and confidence for TFCC lesions.

## Methods

### Study participants

**Fig. 2:**
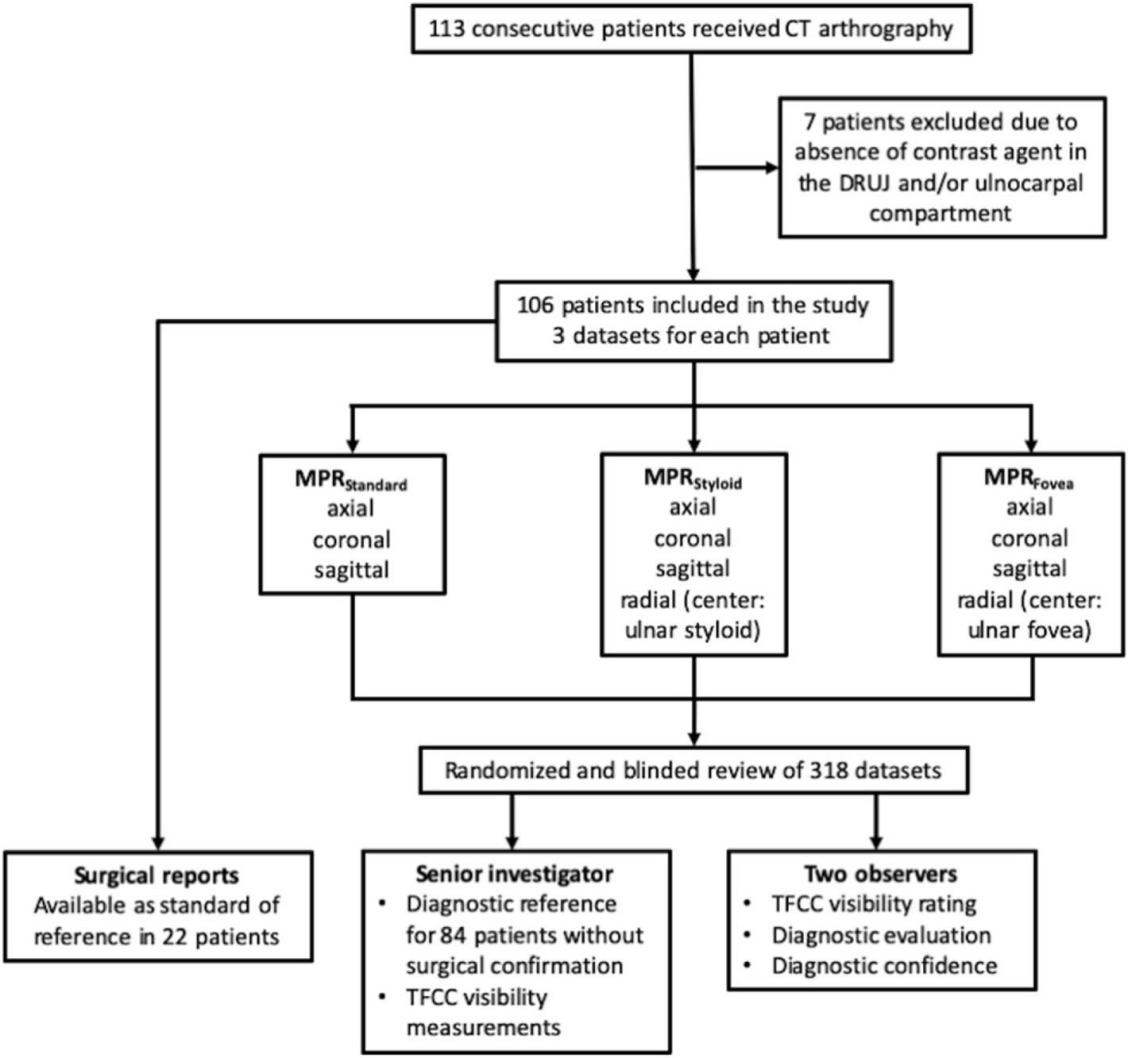
Flow chart for visualization of inclusion / exclusion criteria, study population and individual steps of the review process

Over the course of one year (January 1st to December 31st 2018), 113 consecutive patients of the Department of Hand Surgery at Rhön-Klinikum Campus Bad Neustadt, Germany, received CT imaging after multi-compartment arthrography of the wrist. Seven patients (three women, four men) had to be excluded from the study because no contrast agent was present in the DRUJ and/or ulnocarpal compartment, thus ruling out diagnostic inspection for TFCC injury. Therefore, the final study group consisted of 106 patients, including 49 women and 57 men with mean age of 44.2 ± 15.8 years. Inclusion and exclusion criteria are depicted in Fig. 2. Previous wrist trauma was reported in the far majority of patients (89) with the median time between trauma and CT arthrography being 28 days (interquartile range 5–190 days). As we aimed to assess the importance of radial MPR for any kind of TFCC lesion, irrespective of trauma history, a small number of patients without trauma that received CT arthrography during that time period were also enrolled in the study. The left wrist was examined in 57, the right wrist in 49 patients.

### Wrist arthrography

Board-certified radiologists performed carpal arthrography under fluoroscopic guidance using a contrast medium (Imeron 300, Bracco Imaging, Konstanz, Germany) diluted with sodium chloride for an iodine amount of 150 mg/ml. As multi-compartment arthrography demonstrably provides better diagnostic accuracy for TFCC lesions compared to mono-compartment arthrography [22], all procedures were conducted with the following compartment order: midcarpal joint (for potential scapholunate or lunotriquetral ligament tears; only if suspected), DRUJ, radiocarpal joint.

### CT scan and reconstruction parameters

**Fig. 3:**
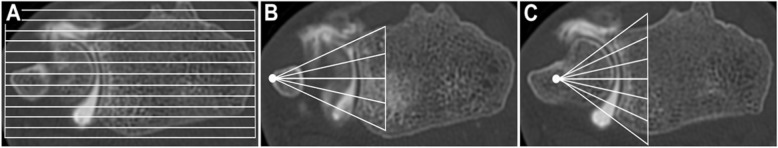
35-year-old man with ulnar-sided wrist pain. Axial planes of the distal radioulnar joint illustrate the difference between multiplanar reconstructions after CT arthrography. **a** coronal reconstruction. **b** radial reconstruction with the rotating center atop the ulnar styloid process. **c** radial reconstruction with the rotating center in the ulnar fovea

Following fluoroscopy-guided arthrography, CT scans were performed with the patient in prone position, the afflicted arm extended above the head and the wrist in pronation (“superman position”). Axial images were acquired with slice thickness and increment of 0.6 mm and 0.3 mm, respectively, using a commercially available multidetector CT scanner (Optima 660, GE Healthcare, Chicago, Illinois, USA). Reference tube voltage of 120 kVp and reference tube current of 150 mAs were applied. Detector collimation was 64 × 0.6 mm and pitch factor 1.2. In post-processing, coronal and sagittal planes with slice thickness of 1 mm, increment of 0.5 mm, image matrix of 1024 × 1024 pixels and field of view of 60 mm were reconstructed by a radiologic technologist using scanner side software (Advantage Workstation, GE Healthcare). Two additional radial MPR were adjusted to the triangular shape of the TFCC with an angle between images of 2°. The rotating center of one radial MPR was positioned atop the ulnar styloid, while the center of the other angulated MPR was located in the ulnar fovea (Fig. 3). Readers were allowed to change settings for window width and level to their own demands.

### Reference standard

Based on imaging results and clinical examination, 22 patients received arthroscopic exploration of the ulnocarpal compartment. For the corresponding CT arthrographies, the surgical report was used as standard of reference. Wrist arthroscopies were performed in standardized fashion, using carbon dioxide and sodium chloride as media. For intraoperative assessment of TFCC stability, visual evaluation was conducted through a portal between the extensor tendon compartments 3/4. After insertion of a probe through the 4/5-portal, the trampoline test was used to examine the firmness of the TFCC for compressive load [23]. In addition, the hook test was applied to assess the stability of the deep TFCC layer. By inserting the probe underneath the ulnar aspect of the horizontal part of the TFCC and pulling in a radial and distal direction, insufficiencies of the ulnar attachment can be detected if the entire discus is lifted towards the lunate [24, 25]. In case of an isolated proximal lesion, a positive ghost sign can be observed on the foveal aspect of the TFCC [26]. If necessary, additional instruments were inserted, for example to execute the push-off needle test [27]. As recommended by Löw et al., adequate photo and video documentation of arthrographies was performed to enhance intra- and interobserver reliability [28, 29]. In the remaining 84 patients, no surgical exploration was realized based on wrist stability in clinical assessment and imaging results. For these patients, the evaluation of CT arthrography by a senior musculoskeletal radiologist with 35 years of wrist imaging experience (RS) was used as reference standard. To provide an additional measurement of TFCC visibility, the senior investigator quantified the percentage of CT slices depicting the full extent of the TFCC in coronal and radial planes for each CT arthrography.

### Observer analysis

For each patient, three datasets were assembled with the first set (MPR_Standard_) containing only the three radiological standard planes (axial, coronal, sagittal), the second set (MPR_Styloid_) including standard planes and the styloid-centered radial MPR and the third set (MPR_Fovea_) comprising standard planes and the fovea-centered angulated MPR. Two radiologists with nine (TG; reader 1; R1) and five (KL; reader 2; R2) years of experience in musculoskeletal imaging analyzed all datasets in randomized and blinded fashion with dedicated PACS software (Merlin, Phönix-PACS, Freiburg, Germany). For each dataset, readers were asked for their assessment of TFCC continuity. In case of suspected discontinuity, they should also categorize the lesion according to the classifications of Palmer and Atzei and state their diagnostic confidence using a five-point Likert scale (5 = total confidence; 4 = high confidence; 3 = moderate confidence; 2 = slight confidence; 1 = little to no confidence). Furthermore, observers evaluated the visibility of the deep and superficial TFCC layer as well as the interposed connective tissue known as the “ligamentum subcruentum” on a three-point scale (3 = good visibility; 2 = moderate visibility; 1 = little to no visibility) for each set.

### Statistics

We used dedicated software (SPSS Statistics Version 23.0 for Mac, IBM, Amonk, New York, USA) to perform statistical analyses. Categorical variables (e.g. scale results) are presented as frequencies and percentages with medians and interquartile ranges, whereas normally distributed data is presented as means ± standard deviation (SD). Normal distribution of continuous variables was assessed using Kolmogorov-Smirnov tests. Wilcoxon signed rank tests were used to compare paired nonparametric variables. Comparison of classification functions (e.g. sensitivity, specificity) for different MPR was conducted with the McNemar test. To determine interrater agreement, Cohen’s weighted kappa values were calculated for each dataset [30]. 휅 values were interpreted according to Landis and Koch (1.00–0.81 = almost perfect agreement; 0.80–0.61 = substantial agreement; 0.60–0.41 = moderate agreement; 0.40–0.21 = fair agreement; 0.20–0.00 slight agreement; < 0.00 poor agreement) [31]. *P* values ≤0.05 were considered to indicate statistical significance.

## Results

### Evaluation of TFCC depiction

The median percentage of CT planes displaying the full extent of the deep TFCC layer was 32.0% (interquartile range 24.0–41.0%) for MPR_Standard_, 87.0% (74.0–100.0%) for MPR_Styloid_ and 98.5% (88.3–100.0%) for MPR_Fovea_. The superficial layer of the TFCC was fully visible in 34.5% (29.0–41.8%) of coronal images, whereas 100.0% (91.0–100.0%) of MPR_Styloid_ slices and 94.0% (73.3–100.0%) of MPR_Fovea_ planes depicted its entirety.

Table 1 illustrates reader’s subjective evaluation of TFCC component depiction in CT arthrography. The deep TFCC layer was less visible in standard planes compared to the styloid-centered (R1/R2, *p* < 0.001) and fovea-centered (R1/R2, p < 0.001) MPR. Discernibility of the superficial TFCC layer was also superior for MPR_Styloid_ (R1, *p* = 0.007; R2, *p* < 0.001) and MPR_Fovea_ (R1/R2, p < 0.001). Visibility of the ligamentum subcruentum was good in only 27.4/22.6% of MPR_Standard_, 31.1/31.1% of MPR_Styloid_ and 34.0/31.1% of MPR_Fovea_ images. However, as with both TFCC layers, the styloid-centered (R1, *p* = 0.005; R2 p < 0.001) and fovea-centered (R1, *p* = 0.002; R2, *p* = 0.006) radial planes provided superior differentiability compared to the standard MPR.

**Table 1:** Differentiability of TFCC components in standard planes and radial plane view. Evaluation by two readers using a three-point Likert scale (3 = good visibility, 2 = moderate visibility, 1 = little to no visibility). Scale results are reported as frequencies (percentages)

Visibility	Deep layer of TFC			Superficial layer of TFC			Ligamentum subcruentum		
	MPR_Standard_	MPR_Styloid_	MPR_Fovea_	MPR_Standard_	MPR_Styloid_	MPR_Fovea_	MPR_Standard_	MPR_Styloid_	MPR_Fovea_
**Reader 1**									
**3**	64 (60.4)	80 (75.5)	87 (82.1)	72 (67.9)	82 (77.4)	90 (84.9)	29 (27.4)	33 (31.1)	36 (34.0)
**2**	30 (28.3)	20 (18.9)	13 (12.3)	27 (25.5)	19 (17.9)	12 (11.3)	14 (13.2)	18 (17.0)	15 (14.2)
**1**	12 (11.3)	6 (5.7)	6 (5.7)	7 (6.6)	5 (4.7)	4 (3.8)	63 (59.4)	55 (51.9)	55 (51.9)
**Reader 2**									
**3**	53 (50.0)	73 (68.9)	81 (76.4)	62 (58.5)	88 (83.0)	86 (81.1)	24 (22.6)	33 (31.1)	33 (31.1)
**2**	31 (29.2)	23 (21.7)	16 (15.1)	33 (31.1)	13 (12.3)	15 (14.2)	14 (13.2)	13 (12.3)	12 (11.3)
**1**	22 (20.8)	10 (9.4)	9 (8.5)	11 (10.4)	5 (4.7)	5 (4.7)	68 (64.2)	60 (56.6)	61 (57.5)

TFC Triangular fibrocartilage (ulnocarpal disc), *DL* Deep layer of the peripheral TFC, *SL* Superficial layer of the peripheral TFC, *MPR*_*Standard*_ Standard multiplanar reconstructions (axial, coronal, sagittal planes), *MPR*_*Styloid*_ Radial multiplanar reconstruction with rotating center atop the ulnar styloid, *MPR*_*Fovea*_ radial multiplanar reconstruction with the rotating center in the ulnar fovea

### TFCC findings in CT arthrography

In 106 patients, 92 ulnocarpal complexes presented partial or complete tears that could be categorized according to Palmer’s classification. Therefore, the total prevalence of TFCC lesions in the study group was 86.8%. Forty seven patients (44.3%) presented peripheral lesions (Palmer 1b and 1c), while 73 CT arthrographies (68.9%) showed central TFCC alterations (Palmer 1a, 1d and 2a–e). Twenty eight patients (26.4%) exhibited simultaneous central and ulnar-sided lesions.

**Fig. 4:**
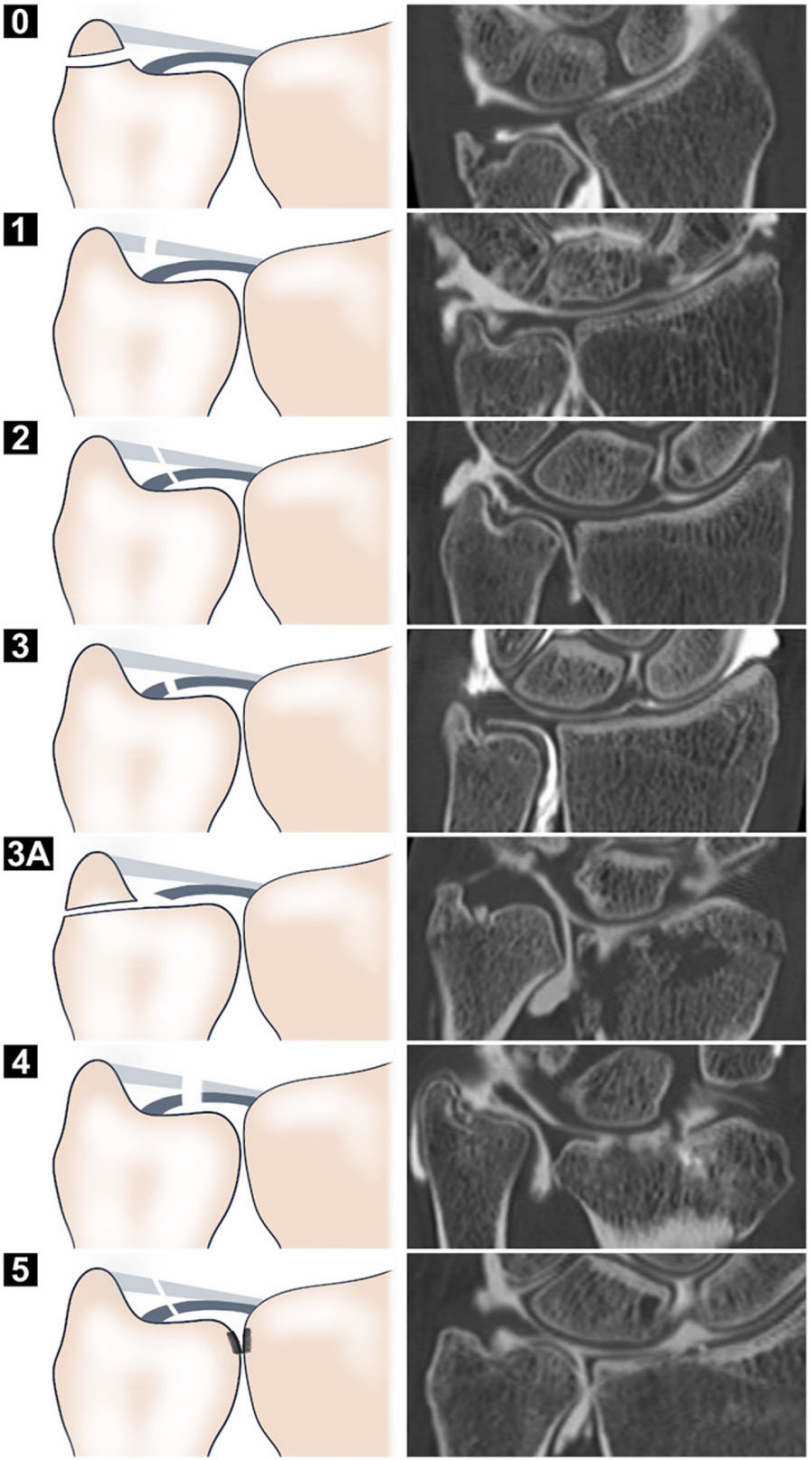
Left side: Schematic illustration of the Atzei classification for peripheral lesions of the triangular cartilage complex (TFCC). Right side: Radially reconstructed CT images from different patients depicting the corresponding ulnar-sided TFCC pathologies. Additional central discus alterations are present in the images for stages 3A and 5

Using Atzei’s classification of peripheral TFCC alterations (Fig. 4), 56 patients (52.8%) presented corresponding lesions. Table 2 depicts the exact categorization of TFCC lesions according to Palmer’s and Atzei’s classification systems.

**Table 2:** Categorization of TFCC lesions according to Palmer’s and Atzei’s classification. For Palmer’s classification, simultaneous peripheral and central lesions are possible, while Atzei’s classification is exclusive to ulnar-sided pathologies. Results are reported as frequencies (percentages)

Palmer	Lesions	Description	Atzei	Lesions	Description
**1a**	11 (10.4)	Central perforation	**0**	10 (9.4)	Isolated styloid fracture
**1b**	46 (43.4)	Ulnar avulsion	**1**	13 (12.3)	SL tear
**1c**	1 (0.9)	Ulnocarpal ligament injury	**2**	7 (6.6)	Complete peripheral tear
**1d**	2 (1.9)	Radial avulsion	**3**	11 (10.4)	DL tear
**2a**	24 (22.6)	TFC wear	**3A**	6 (5.7)	Avulsion fracture of DL insertion
**2b**	24 (22.6)	TFC wear + chondromalacia	**4**	3 (2.8)	Non-repairable peripheral tear
**2c**	7 (6.6)	TFC perforation + chondromalacia	**5**	6 (5.7)	Peripheral tear + DRUJ arthritis
**2d**	3 (2.8)	Palmer 2c + LTL tear			
**2e**	2 (1.9)	Palmer 2d + ulnocarpal arthritis			
**No lesion**	14 (13.2)		**No lesion**	50 (47.2)	
**Total lesions**	120		**Total lesions**	56	
**Combined**	28 (26.4)	Peripheral and central lesions			

*TFCC* Triangular fibrocartilage complex, *TFC* Triangular fibrocartilage (ulnocarpal disc), *LTL* Lunotriquetral ligament, *SL* Superficial layer of the peripheral TFCC, *DL* Deep layer of the TFCC, *DRUJ* Distal radioulnar joint

### Detection of TFCC lesions for standard and radial MPR

For the 73 central TFCC alterations according to Palmer’s classification, specificity, sensitivity and accuracy were consistently very high with no substantial difference between radial and standard reconstructions. Contrarily, observer ratings for the 47 peripheral Palmer lesions displayed more heterogenous classification functions. While specificity was very good for all datasets, sensitivity and accuracy were considerably higher with the addition of the styloid-centered (R1, *p* = 0.004; R2, p = 0.004) and fovea-centered (R1, *p* = 0.039; R2, p = 0.039) radial MPR. No difference was found between datasets including the two radial reconstructions (R1, *p* = 0.688; R2, *p* = 0.500). Accordingly, for the 56 Atzei lesions in the study group, specificity was excellent, irrespective of reconstruction type. In contrast, sensitivity and accuracy were unanimously better for the styloid-centered radial MPR compared to standard planes (R1, *p* = 0.004; R2, *p* = 0.008). For one observer, sensitivity and accuracy of the fovea-centered MPR was also higher than for standard planes alone (R1, *p* = 0.039; R2, *p* = 0.125), while no difference was observed between the two datasets containing radial MPR (R1, *p* = 0.688; R2, *p* = 0.250). Table 3 illustrates statistical measures for Atzei and Palmer lesions. Interobserver agreement was almost perfect for either dataset: MPR_Standard_ (휅=0.876; 95% confidence interval: 0.796–0.956; *p* < 0.001), MPR_Styloid_ (휅=0.894; 0.816–0.972; p < 0.001) and MPR_Fovea_ (휅=0.949; 0.894–1.00; p < 0.001).

**Table 3:** Classification functions for detection of Palmer and Atzei lesions. Indicators of diagnostic accuracy for TFCC lesions in standard planes and radial plane view. Data is reported as percentages (numerator/denominator)

		Reader 1			Reader 2	
	MPR_Standard_	MPR_Styloid_	MPR_Fovea_	MPR_Standard_	MPR_Styloid_	MPR_Fovea_
**Central Palmer lesions (*n*** **= 73)**						
Specificity	97.0 (32/33)	97.0 (32/33)	97.0 (32/33)	93.9 (31/33)	93.9 (31/33)	93.9 (31/33)
Sensitivity	93.2 (68/73)	94.5 (69/73)	94.5 (69/73)	91.8 (67/73)	90.4 (66/73)	90.4 (66/73)
Accuracy	94.3 (100/106)	95.3 (101/106)	95.3 (101/106)	92.5 (98/106)	91.5 (97/106)	91.5 (97/106)
Positive predictive value	98.6 (68/69)	98.6 (69/70)	98.6 (69/70)	97.1 (67/69)	97.1 (66/68)	97.1 (66/68)
Negative predictive value	86.5 (32/37)	88.9 (32/36)	88.9 (32/36)	83.8 (31/37)	81.6 (31/38)	81.6 (31/38)
**Peripheral Palmer lesions (*n*** **= 47)**						
Specificity	96.6 (57/59)	98.3 (58/59)	98.3 (58/59)	93.2 (55/59)	98.3 (58/59)	98.3 (58/59)
Sensitivity	70.2 (33/47)	89.4 (42/47)	85.1 (40/47)	72.3 (34/47)	91.5 (43/47)	87.2 (41/47)
Accuracy	84.9 (90/106)	94.3 (100/106)	92.5 (98/106)	84.0 (89/106)	95.3 (101/106)	93.4 (99/106)
Positive predictive value	94.3 (33/35)	97.7 (42/43)	97.6 (40/41)	89.5 (34/37)	97.7 (43/44)	97.6 (41/42)
Negative predictive value	80.3 (57/71)	92.1 (58/63)	89.2 (58/65)	80.9 (55/68)	93.5 (58/62)	90.6 (58/64)
**Atzei lesions (*n*** **= 56)**						
Specificity	96.0 (48/50)	98.0 (49/50)	98.0 (49/50)	94.0 (47/50)	98.0 (49/50)	98.0 (49/50)
Sensitivity	78.6 (44/56)	94.6 (53/56)	91.1 (51/56)	80.4 (45/56)	94.6 (53/56)	89.3 (50/56)
Accuracy	86.8 (92/106)	96.2 (102/106)	94.3 (100/106)	86.8 (92/106)	96.2 (102/106)	93.4 (99/106)
Positive predictive value	95.7 (44/46)	98.1 (53/54)	98.1 (51/52)	93.8 (45/48)	98.1 (53/54)	98.0 (50/51)
Negative predictive value	80.0 (48/60)	94.2 (49/52)	90.7 (49/54)	81.0 (47/58)	94.2 (49/52)	89.1 (49/55)

*TFCC* Triangular fibrocartilage complex, *MPR*_*Standard*_ Standard multiplanar reconstructions (axial, coronal, sagittal planes), *MPR*_*Styloid*_ Radial multiplanar reconstruction with rotating center atop the ulnar styloid, *MPR*_*Fovea*_ Radial multiplanar reconstruction with the rotating center in the ulnar fovea

### Diagnostic confidence

Readers declared at least high diagnostic confidence (Likert scale values 4 and 5) in the far majority of CT arthrographies. Total confidence (Likert scale value 5), however, was more often stated for datasets containing radial MPR in comparison to only the three standard planes (all p < 0.001). Observers reported total confidence in 54.7%/35.8% (R1/R2) of MPR_Standard_, 80.1%/55.7% of MPR_Styloid_ and 79.2%/60.4% of MPR_Fovea_ (Table 4).

**Table 4:** Diagnostic confidence. Evaluation by two readers using a five-point Likert scale (5 = total confidence, 4 = high confidence, 3 = moderate confidence, 2 = slight confidence, 1 = little to no confidence). Scale results are reported as frequencies (percentages) and median values

		Reader 1			Reader 2	
Confidence	MPR_**Standard**_	MPR_**Styloid**_	MPR_**Fovea**_	MPR_**Standard**_	MPR_**Styloid**_	MPR_**Fovea**_
**5**	58 (54.7)	85 (80.1)	84 (79.2)	38 (35.8)	59 (55.7)	64 (60.4)
**4**	35 (33.0)	16 (15.1)	16 (15.1)	21 (19.8)	32 (30.2)	26 (24.5)
**3**	10 (9.4)	5 (4.7)	6 (5.7)	34 (32.1)	14 (13.2)	14 (13.2)
**2**	3 (2.8)	0 (0.0)	0 (0.0)	10 (9.4)	1 (0.9)	2 (1.9)
**1**	0 (0.0)	0 (0.0)	0 (0.0)	3 (2.8)	0 (0.0)	0 (0.0)
**Median**	5	5	5	4	5	5

*TFCC* Triangular fibrocartilage complex, *MPR*_*Standard*_ Standard multiplanar reconstructions (axial, coronal, sagittal planes), *MPR*_*Styloid*_ Radial multiplanar reconstruction with rotating center atop the ulnar styloid, *MPR*_*Fovea*_ Radial multiplanar reconstruction with the rotating center in the ulnar fovea

## Discussion

For this study, we retrospectively analyzed 106 CT arthrographies of the wrist, focusing on the detection of TFCC pathologies and diagnostic confidence for standard reconstructions and radial plane view. Therefore, we compared datasets containing the three radiological standard planes (axial, coronal, sagittal; MPR_Standard_) with datasets comprising additional radial reconstructions with the rotating center positioned either atop the ulnar styloid process (MPR_Styloid_) or in the ulnar fovea (MPR_Fovea_).

Functioning as the main stabilizer of the distal radioulnar joint, detection and categorization of TFCC injuries is important for deciding whether surgery is necessary. Considering the limited applicability of DRUJ arthroscopy if the deep attachment of the TFCC is intact, non-invasive diagnostics should always precede its surgical exploration. Furthermore, being a highly specialized procedure, the availability of dedicated arthroscopic evaluation of the DRUJ and ulnocarpal compartment is mostly limited to a small number of centers and does not represent the diagnostic standard for TFCC lesions. Not every TFCC lesion can be identified in contrast-enhanced MRI, though, often requiring wrist arthrography with subsequent 3D imaging to evaluate the extent of TFCC discontinuity [16]. The advantages of injecting contrast agent into different compartments of the wrist have been shown for interosseous ligament and ulnocarpal complex injuries before [32–34]. Joint distension improves the visibility of anatomical structures [35] and with regard to the TFCC, facilitates the differentiation between its deep and superficial layer [17, 18]. Moreover, the presence of contrast agent allows for direct visualization of cartilaginous or ligamentous defects [36]. Our results for specificity, sensitivity and accuracy are concordant with a meta-analysis of 28 studies by Treiser et al. [37], confirming that overall diagnostic accuracy is generally higher for central compared to peripheral TFCC alterations. However, the detection rate of peripheral TFCC discontinuity was considerably better with the addition of radial plane view and almost reached the diagnostic accuracy for central lesions in this study. A possible explanation suggested by our results might be the limited visibility of the ulnar-sided TFCC in radiological standard planes due to its complex three-dimensional shape. While only one third of coronal CT planes depicted the entire length of the TFCC (mostly the central part), almost every image of both radial MPR displayed its full extent. Therefore, the number of slices depicting a lesion might be higher in radial plane view, which could particularly help identifying partial lesions of the dorsal and palmar radioulnar ligament. Accordingly, two radiologists deemed the visibility of the TFCC’s deep and superficial layer as well as the visualization of the interpositioned ligamentum subcruentum superior with addition of either radial MPR in this study. As the rotating centers for the radial plane view were positioned congruently with the two layers’ ulnar insertions, we assumed that visibility of each layer would be superior for the respective radial MPR. However, this was only true for the deep TFCC layer, which was favorably depicted in the fovea-centered radial plane view.

### Limitations

Several limitations have to be acknowledged regarding this study. Firstly, some of the detected central TFCC alterations might be attributed to subclinical degenerations, which are considered physiological in individuals aged 30 years and older [38–40]. In contrast, peripheral lesions, are mostly associated with traumatic injuries. Therefore, the improved accuracy provided by radial plane view for the detection of ulnar-sided TFCC lesions is of high clinical relevance. Secondly, partial and complete TFCC tears were not differentiated in this study following the classifications of Palmer [9] and Atzei [11]. As both classifications have originally been introduced for surgical assessment of TFCC injuries, their radiological application has to be interpreted with caution. The dorsal and palmar capsular fixations of the TFCC were also not evaluated in this study, as their clinical impact has not been fully described to date. Finally, surgical confirmation was only available in 22 patients, requiring an additional standard of reference for this study. This limitation can be partially attributed to the importance of CT arthrography for ulnocarpal complex assessment, as arthroscopic exploration was not performed if the DRUJ was clinically stable and the deep fibers of the TFCC proved to be intact in CT arthrography. Degenerative alterations of the central cartilage and isolated lesions of the superficial TFCC attachment do not necessarily require surgical treatment to maintain stability, subsequently restricting the number of interventions. No clinical follow-up was available for patients that only received diagnostic imaging but did not undergo any surgical procedure at our institution.

## Conclusions

Addition of radial multiplanar reconstructions is recommended for evaluation of triangular fibrocartilage complex injuries in CT arthrography of the wrist. While central TFCC alterations can be reliably detected using the three radiological standard planes (axial, coronal, sagittal), diagnostic accuracy for the clinically relevant peripheral lesions is substantially higher with ancillary reconstructions adjusted to the complex three-dimensional anatomy of the ulnar-sided TFCC. Furthermore, superior differentiability of the deep and superficial TFCC layer in radial plane view improves overall diagnostic confidence.

## Data Availability

The datasets generated and/or analyzed during this study are not publicly available as CT data and DICOM headers contain patient information. Data can be obtained on reasonable request from the corresponding author.

## Abbreviations

TFCC: Triangular fibrocartilage complex
TFC: Triangular fibrocartilage (ulnocarpal disc)
DRUJ: Distal radioulnar joint
MPR: Multiplanar reconstruction
MDCT: Multi-detector computed tomography
DL: Deep layer of the TFC
SL: Superficial layer of the TFC
